# Development of Dietary Patterns Spanning Infancy and Toddlerhood: Relation to Body Size, Composition and Metabolic Risk Markers at Three Years

**DOI:** 10.3934/publichealth.2015.3.332

**Published:** 2015-07-27

**Authors:** Louise BB Andersen, Christian Mølgaard, Katrine T Ejlerskov, Ellen Trolle, Kim F Michaelsen, Rasmus Bro, Christian B Pipper

**Affiliations:** 1 Department of Nutrition, Exercise and Sports, University of Copenhagen, Nørre Allé 51, DK-2200 Copenhagen N, Denmark; 2 National Food Institute, Technical University of Denmark, Mørkhøj Bygade 19, DK-2860 Søborg, Denmark; 3 Department of Food Science, University of Copenhagen, Rolighedsvej 26, DK-1958 Frederiksberg, Denmark; 4 Department of Public Health, University of Copenhagen, Øster Farimagsgade 5, DK-1014 Copenhagen K, Denmark

**Keywords:** dietary patterns, risk markers, tracking, toddlerhood, principal component analysis, longitudinal

## Abstract

Little is known about the development of dietary patterns during toddlerhood and the relation to growth and health. The study objective was to characterise the development of dietary patterns from 9–36 mo of age and investigate the association to body size, body composition and metabolic risk markers at 36 mo. Food records were filled out at 9, 18 and 36 mo of age (n = 229). Dietary patterns were identified by principal component analysis (PCA). Three dietary patterns were identified: *Transition Food*, *Healthy Food* and *Traditional Food*. The course of development in dietary patterns from 9–36 mo indicated tracking for a relatively large group of participants in the three patterns. *Transition Food* and *Healthy Food* were associated with some of the investigated outcomes. Children with lower adherence to the *Transition Food* pattern than average at 18 and 36 mo irrespectively of intake at 9 mo had higher BMI z-scores at 36 mo. Similar trend was identified for higher fat mass indices. Children with lower adherence to the *Healthy Food* pattern than average at all three ages compared to children with higher adherence to the *Healthy Food* pattern at the first two registrations, 9 and 18 mo had higher total cholesterol and LDL. Hence, this could represent undesirable development of dietary patterns in toddlers. In conclusion, development of dietary patterns can be exploratory characterised by PCA and related to potential cardiovascular risk markers in toddlers even within a relatively homogeneous population with a high socioeconomic status. The tracking of dietary patterns from 9 mo of age indicates a need for early and sustained promotion of healthy diets.

## 1. Introduction

Within the first years of life, infants and toddlers substantially change their sources of nutrients from a milk-based diet to the same diet as the rest of the family. This is the most extensive dietary change made throughout life, and furthermore this is the period of the most rapid growth. The first years of life are a window of opportunity because early nutrition and growth seem to have long-lasting consequences for the risk of obesity and cardiovascular diseases [1]. Furthermore, some evidence shows that early dietary patterns seem to track into later childhood and adulthood [2]–[4].

The consequences of breastfeeding versus formula-feeding is widely investigated [5], while knowledge about complementary feeding and a toddler's diet in relation to current body size and later health is still sparse [6]. Going from milk to the family's food increases the complexity of the dietary pattern. In general, it is realised that the relation between diet and health cannot be fully characterised by a few nutrients or foods [7],[8]. Instead, a dietary-pattern approach based on foods eaten at different ages during infancy and toddlerhood comprising the whole diet might grasp more of this complexity. The most common method to summarise the complexity of dietary patterns is the principal component analysis (PCA) [9]. PCA is an explorative, data-driven method which allows inclusion of many highly correlated foods simultaneously. PCA uses the correlations between the large numbers of dietary variables to identify latent dimensions in the data [10]. However, few studies have characterised dietary patterns using PCA in young children from high income countries [4];[11]–[15]. To the best of our knowledge only three of these; an Australian study [11] and the two British studies Southampton Women Study (SWS) [4] and Avon Longitudinal Study of Parents And Children (ALSPAC) [12] have investigated dietary patterns longitudinally in infancy and toddlerhood. Brazionis and colleagues investigated in ALSPAC the development of dietary patterns at three ages (6, 15 and 24 mo, n = 2169) and found two consistent dietary patterns over time [12]. The dietary patterns were not examined for relations with anthropometry and metabolic risk factors; neither was individual tracking of dietary patterns investigated. However, in a later publication the relation between the dietary patterns and blood pressure at 7 years of age was investigated [16]. They found that a less healthy diet at 2 years was associated with a higher blood pressure at 7 years. The relation between dietary patterns and body size or composition has only been investigated for dietary patterns at one time point during infancy and toddlerhood [11],[17], not including the effect of development of dietary patterns across different ages. The Australian study investigated dietary patterns separately at 14 and 24 mo and did not find an association between dietary patterns and BMI z-scores at 14 (n = 467) and 24 (n = 404) months [11]. SWS found a positive association between skinfold thickness at 12 months and an infant guideline pattern at 6 months, but not with dietary patterns at 12 months of age (n = 1308) [17]. Furthermore, to our knowledge, the relation to metabolic risk markers is yet unexplored.

Firm characterisations of the development of dietary patterns during the first years of life as well as knowledge about the impact on key anthropometric and metabolic outcomes are important to support health guidance. The aim of this study was to characterise the development in dietary patterns from 9 to 36 mo of age and to explore the association between the development in dietary patterns and body size, body composition and metabolic risk markers at 36 mo.

## 2. Materials and Method

### 2.1. Study design and participants

Data were from a Danish longitudinal observational cohort study (SKOT I; Danish abbreviation of small children's diet and wellbeing) which monitored children at 9 (+/-14 days), 18 (+/-30days) and 36 (+/-90 days) mo of age from year 2007 to 2010 and was previously described by Madsen and colleagues [18]. Mailed invitations were sent to 2,211 families randomly selected from the National Danish Civil Registry in the capital area. Inclusion criteria were singleton infants born ≥ 37 weeks of gestation without diseases expected to affect growth or food intake and with Danish speaking parents. Anthropometric assessments were performed at three visits accompanied by instructions in dietary assessment, background questionnaires and parental BMI assessments. The study protocol was approved by The Committees on Biomedical Research Ethics for the Capital Region of Denmark (H-KF-2007-0003). Informed written consent was obtained from all parents.

### 2.2. Dietary data

The diet of each child was recorded by parents after an oral and written introduction for seven consecutive days at 9, 18 and 36 mo of age using a seven day food record method. This method has been shown to be valuable to estimate dietary intake in this age groups when comparing with double-labelled water [19]. Portion sizes were estimated with household measures and a photo booklet, and were noted in a pre-coded food record. Intake of food items, energy and nutrients were calculated for each participant using the software General Intake Estimation System (GIES, version 1.000d, developed at National Food Institute, Technical University of Denmark) and the Danish Food Composition Databank (version 7; Søborg; www.Foodcomp.dk, visited May 2011) as described previously [19]. Quality control was carried out by trained research staff before data were entered in the database.

Possible over- and under-reporters of energy intake were identified based on Goldberg cut-off [20] using the Schofield equations [21] to estimate basal metabolic rate at 18 and 36 mo. At 9 mo possible over- and under-reporters of energy intake were identified based on the estimated daily energy requirement of 338kJ/kg Body Weight(BW)/day for both genders as an average between 6 and 12 mo estimates [22] and cut-off values of +/-46% [23].

The food group variables for the PCA model were the same for all three ages selected based on nutritional knowledge trying to cover most aspects of the official recommendations, nutrition evidence and typical toddler diet in Denmark. In total the PCA was based on 25 food groups which are named with a short, compressed description, such as ‘FatsAnimal’ and ‘SugaryDrink’. The mean intake (g/day) of all food groups, except for breast milk, was divided by total body weight (BW, kg) for each participant at each age to make the contribution of each food to the whole diet comparable across different ages and requirements. Intake of breast milk was estimated as number of breastfeeding sessions per day according to interview with parents (in the categories: <1, 1–2, 3–5, 6–8, >9 times/day) and recoded as the mean of each interval. These categories (feedings/day) were used in the PCA while an estimate of 99 g/feeding [24] was used to calculate g/kg BW/day for the bar plot of foods and in the calculation of total energy intake.

### 2.3. Anthropometry

Weight was measured without clothes to the nearest 0.1 kg using a digital scale (At 9 mo: Sartorius IP 65; Sartorius AG, Göttingen, Germany; at 18 mo: Lindeltronic 8000, Samhall Lavi AB, Kristianstad, Sweden; at 36 mo: Tanita WB-100MA, Tanita Corporation, Tokyo, Japan). Height at 36 mo was measured by a stationary digital height measurer (235 Heightronic Digital Stadiometer), which made readings to the nearest 0.01 cm. Height was performed in triplicates, and the average was used in analysis. BMI (kg/m^2^) was calculated from weight and height. Child height and BMI were converted to z-scores using the WHO growth standard as reference and the software program WHO Anthro 2005 [25].

### 2.4. Blood samples

Blood triacylglycerol, glucose, insulin, total cholesterol, low density lipoprotein (LDL), high density lipoprotein (HDL), insulin-like growth factor 1 (IGF-I) and insulin-like growth factor binding protein 3 (IGFBP3) were measured in a blood sample (plasma, except glucose which were analysed in full blood) taken at the 36 mo visit after a two hour fast. Mean fasting time (±SD) was 174 ± 36 minutes with the exception of 29 children, who fasted from the night before the examination. Composition of the last meal before fasting was recorded and analysed using the software programme Dankost (version 3000, Dankost Ltd, Copenhagen, Denmark). Insulin, IGF-I, and IGFBP3 were determined on an Immulite 1000 analyser (Diagnostic Products Corporation, USA); glucose was determined in EDTA on HemoCue (HemoCue Denmark, Denmark); and total cholesterol, LDL, HDL and triacylglycerol were determined on a Pentra 400 analyser (HORIBA ABX, 34184 Montpellier, Cedex 4, France). To estimate the IGF-I/IGFBP3 molar ratio, which is an expression of the bioactive fraction of IGF-I, the following conversion equivalents were used: 1 ng/mL IGF-I = 0.133 nM IGF-I and 1 ng/mL IGFBP3 = 0.033 nM IGFBP3. More details about blood sampling have been published elsewhere [18],[26].

### 2.5. Body composition

Predictive equations for fat free mass and fat mass have previously been generated using bioelectrical impedance (Quantum III, RJL Systems, Michigan, USA), and height and weight from this cohort at 36 mo [27]. This was used to calculate fat free mass index (FFMI) as fat free mass/height^2^ (kg/m^2^) and fat mass index (FMI) as fat mass/height^2^ (kg/m^2^).

### 2.6. Background questionnaire and parental BMI

A background questionnaire at 9 mo included questions about parental age at child's birth, total household income, educational level of parents (updated at 36 mo) and parity. Total household income was divided into < 800,000 DKK (∼145,000 US$) or unknown and > 800,000 DKK per year. The mean household income for a Danish family with two parents and two children is 684,000 DKK per year (125,226 US$) [28]. Educational level was divided into more or less than medium academic education and parity was represented as one child or more children. The weight and height of the parents were measured at the 36 mo visit with the same equipment used for the children, and these measures were used to calculate BMI (kg/m^2^). If weight and height measures from the 36 mo visit were not available, then self-reported values from questionnaires were used.

### 2.7. Statistical analyses

Categorical variables are represented as % (n) and groups with or without three complete diet registrations were compared by the chi^2^ test. Continuous variables are represented as mean ± SD (n) and compared between the groups with or without three complete diet registrations by the unpaired *t*-test, together for boys and girls, while the descriptive presentation is divided by gender.

**Figure 1:**
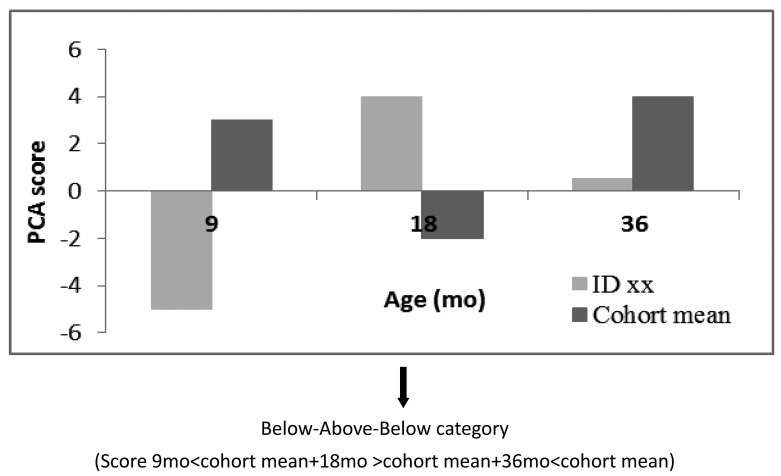
Exemplified illustration of the categorisation system for the fictive child ID xx Child xx ends up in one category for each dietary pattern (not necessarily the same category)

For the identification of dietary patterns, a PCA was carried out including food groups at 9, 18 and 36 mo in long format meaning that the data is structured so that each observation (person at specific time point) is considered as a row in the data matrix, Hence the same person appears in three rows in the matrix with the 25 food groups as variables (columns). This way, a PCA model of the data will provide an estimate of the underlying food patterns that are common over different time points. The score for each observation, on the other hand, will then indicate how much the person adhere to a particular dietary pattern at a particular time point. Thus, by assuming that a general set of latent dietary patterns exist over time, the dynamics of these dietary patterns can be monitored in the scores as a function of time. To normalise data for the PCA they were centred and scaled to unit variance (auto-scaling) [29]. Orthomax, simplimax, verimax and manual rotation were tried but not used, since it did not optimise interpretation of the dietary patterns. Neither did it change the overall interpretation of the dietary patterns to exclude children with the highest total energy intake (> +3 SD) or exclude children partly breastfed at 9 mo whereby they were kept in the model. Naming of dietary patterns was based on subjective assessments of food groups with highest loadings within each principal component meanwhile taking the information of the whole gradient of the dietary pattern into account based on interpretability instead of an arbiter cut-off defining highest. The number of principal components was selected based on the first and largest change in the scree plot [30]. The score variables from the PCA for each principal component were changed into categorical variables; specifically, each child was categorised as above or below the mean score of the cohort for the given age. These three age-specific ranks of the child were combined across ages and placed the child in one of eight categories of development (referred to as “categories of development in dietary patterns”) for each dietary pattern summarising the development in dietary patterns from 9 to 36 mo. The categorisation method is exemplified by the category Below-Above-Below (BAB) in **Figure 1**. Data were analysed in MATLAB R2010b using PLS_Toolbox version 7.3.1. Tracking of dietary patterns across the three ages (9, 18, 36 mo) was evaluated by a comparison of the actual observed number of children with the theoretical calculated number expected if there was no tracking. The number expected if there was no tracking is theoretically calculated as p_9mo_*p_18mo_*p_36mo_*n for the category Above-Above-Above (AAA) and as (1-p_9mo_)*(1-p_18mo_)*(1-p_36mo_)*n for the category Below-Below-Below (BBB), where p = proportion of children above mean, and n = total sample size. If the observed number is higher than the theoretically calculated number the individual adherence to a dietary pattern at different time points are not independent and can be interpreted as tracking.

The relation between different categories of development in dietary patterns and body size (height z-score, BMI z-score), body composition (FMI, FFMI) and metabolic risk markers (glucose, insulin, total cholesterol, HDL, LDL, triacylglycerol, IGF-I, IGFBP3 and IGF-I/IGFBP3) were analysed by ANCOVA adjusted for potential confounders. All analyses were adjusted for sex, parity, parental BMI, parental age, parental education level and household income. Metabolic risk markers were further adjusted for fasting time and energy content in last meal. In ANCOVA analysis, all variables of categories of development in dietary patterns (equal to the number of selected principal components/dietary patterns) were included in the initial model, and backward stepwise elimination was used to identify which of the dietary patterns were significant. Post hoc pairwise comparisons of the eight categories within a dietary pattern were adjusted for multiple testing using the single step method [31]. All statistical tests were conducted in the statistical programming environment R version 3.0.2 (www.r-project.org). All p-values were evaluated at a 5% significance level.

## 3. Results

### 3.1. Subject characteristics

In the SKOT I cohort, 330 children were enrolled, including one child who was excluded before analysis because of late manifestation of a severe chronic disorder. More information about dropouts has been published previously [18]. The number of children with dietary data available was n = 307 at 9 mo, n = 267 at 18 mo and n = 240 at 36 mo all of which were included in the PCA. Only complete dietary cases, meaning children with usable dietary records from all three ages (n = 229, 50% girls), were included in the formation of categories of development in dietary patterns from 9 to 36 mo. Weights at 9 mo (p = 0.03) and 18 mo (p = 0.03) were significantly higher for children without three times of dietary records compared to children with all three dietary records, but these were the only characteristics shown in **Table 1** that were significantly different between children with and without complete dietary data.

**Table 1:** Characteristics of children with complete diet data at 9, 18 and 36 months divided by gender

Child	Girls^a^	Boys^a^
Exclusive breastfeeding, mo	3.7±2.0(115)	3.8±2.0(113)
***9months***		
Examination age, mo	9.1±0.3(115)	9.1±0.3(114)
Weight, kg	8.65±0.85(115)	9.34±0.95(114)
Length, cm	70.72±2.16(115)	72.77±2.33(114)
BMI, kg/m^2^	17.3±1.5(115)	17.6±1.5(114)
***18months***		
Examination age, mo	18.0±0.6(115)	18.0±0.6(113)
Weight, kg	10.77±1.05(115)	11.50±1.12(114)
Length, cm	80.82±2.35(112)	82.73±2.84(114)
BMI, kg/m^2^	16.5±1.4(112)	16.8±1.3(114)
***36months***		
Examination age, mo	36.4±1.0(113)	36.5±1.2(113)
Weight, kg	14.29±1.43(115)	14.88±1.53(114)
Height, cm	94.96±3.12(113)	96.55±3.58(113)
BMI, kg/m^2^	15.9±1.2(113)	16.0±1.1(113)
**Parents and Household**		
Age, mother at birth, y	32±5(115)	31±4(113)
Age, father at birth, y	34±5(114)	34±6(113)
BMI, mother, kg/m^2^	24.0±3.9(115)	24.1±4.4(114)
BMI, father, kg/m^2^	26.1±3.8(114)	25.3±3.2(112)
Education mother		
At least medium academic education	76 (115)	75(114)
Education father		
At least medium academic education	68(111)	60(110)
Parity >1 at 9 mo	40(114)	41(113)
Household income >800,000DKK/y^b^	38(115)	31(113)

^a^ Education, household income and parity represented as % (n).

The rest of the variables are represented as mean±SD (n).

^b^ Information collected in 14 categories comprising intervals with 50,000DKK in each starting from “Below 200,000DKK. Last category “Above 800,000DKK included 35% of the participants. Each of the other categories included less than 8 % of the participants. Mean and SD for the left endpoint of each of the 14 intervals were 611,601DKK and 205,809DKK respectively.

**Figure 2:**
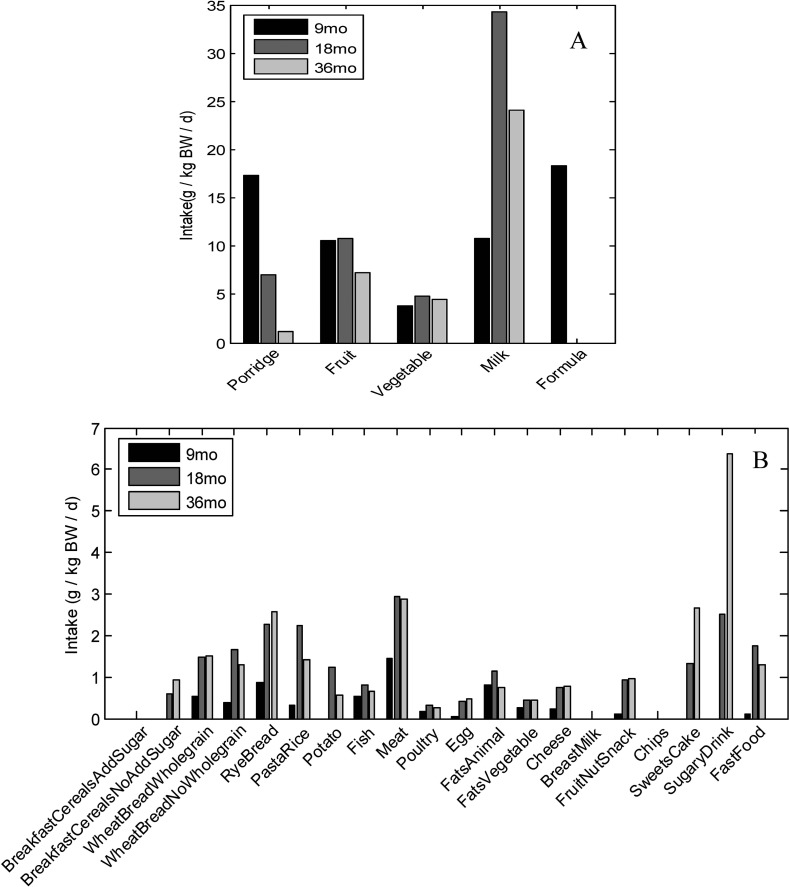
Median intake of food groups **A**: Foods of highest intake. **B**: Foods of lower intake. BW: Body weight. n_9mo_ = 307, n_18mo_ = 267, n_36mo_ = 240 except for *BreastMilk*: n_9mo_ = 310, n_18mo_ = 289, n_36mo_ = 270. Food groups with median intake = 0 illustrate that less than 50% of the children had an intake of this food group.

### 3.2. Dietary patterns

The mean energy intake for children with all three dietary records was 397 kJ/kg BW/day (including estimate of energy from breast milk) at 9 mo, 408 kJ/kg BW/day at 18 mo and 353 kJ/kg BW/day at 36 mo. Of these children 1%, 4% and 15% were possible under-reporters and 16%, 6% and 1% were possible over-reporters at 9, 18 and 36 mo respectively. All these children were included in the analysis, as no major changes were observed in dietary patterns by exclusion. Large variations were seen in the percentage of children eating different foods in the record periods (**Table 2**). WheatBreadNoWholegrain, RyeBread, Fruit, Vegetable, Fish, Meat, FatsAnimal, Cheese and Milk were eaten by 75% or more at all three ages. Other food groups were only eaten by a fraction of children within the recording period, e.g. 19% received SugaryDrink at 9 mo. However, the percentage of children drinking SugaryDrink increased steeply and was 94% at 36 mo (of these e.g. 70% drank juice and 34% drank soda. Yet, the same child might contribute to both percentages). Likewise, a steep increase in the number of children eating FastFood, SweetsCake, Chips and FruitNutSnack were seen from 9 to 36 mo. An overview of the median intake of the different food groups is seen in **Figure 2**. Vegetable, Fruit and various types of milk constitute the biggest weight fraction of the diet at all ages. Infant formula was the main milk at 9 mo while cow's milk nearly constituted the total milk intake at 18 and 36 mo. Porridge was a common food at 9 mo and faded out until 36 mo of age. The median intake of SweetsCake and SugaryDrink was highest at 36 mo.

**Figure 3:**
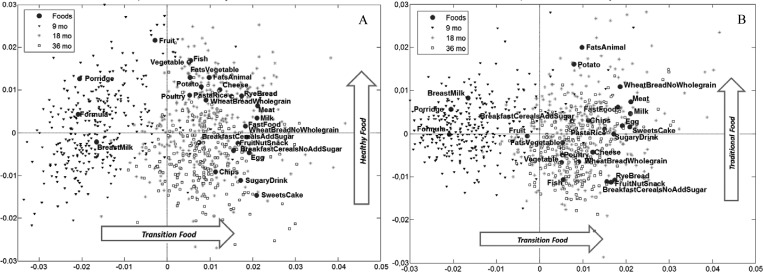
Dietary patterns in the SKOT I cohort **A:** PCA bi-plot of the *Transition Food* and *Healthy Food* patterns. **B**: PCA bi-plot of the *Transition Food* and *Traditional Food* patterns. The foods causing the patterns are indicated with •. Participants (scores) are indicated with ◂ at 9 mo, * at 18 mo and □ at 36 mo. Foods close to each other are correlated while participants placed close to a certain food variable (loading) have a high intake of this food and a lower intake of foods far away, relative to the rest of the participants in the SKOT I cohort. Input variables for the PCA was intake of foods (g/kg BW/day), n_9mo_ = 307, n_18mo_ = 267, n_36mo_ = 240 except for *BreastMilk* (feedings/day): n_9mo_ = 310, n_18mo_ = 289, n_36mo_ = 270. Note that the scale shown on the axes in this plot is arbitrary because the scores and loadings have been individually scaled to fit in the same coordinate system. The same *Transition Food* pattern is shown both in A and B.

The general trend from Figure 2 is recognised in the main dietary patterns generated by PCA and visualised in **Figure 3A** & B. Numerical values of the loadings (**Table S1**) and mean scores per age (**Table S2**) are also available in the supplementary material. Figure 3 is a map of variation in intake of food groups between children. Three dietary patterns were identified. The main variation in food groups (18%), reflected in the first dietary pattern named “*Transition Food*”, is as expected the transition from baby food predominant at 9 mo (Porridge, Formula, BreastMilk) to the versatile intake of different more solid foods which can be eaten by the whole family at later stages. Another 8% of the variation in food groups was described in the second component, named “*Healthy Food*”. This dietary pattern describes a gradient of food intake ranging from potentially unhealthy foods, like SweetsCake, SugaryDrink and Chips, with lowest loadings to healthy food groups, like Fruit, Vegetable and Fish with the highest loadings. The third dietary pattern, explaining 5% of variation, was named “*Traditional Food*” because this pattern differentiated children with highest intake of FatsAnimal, Potato, Meat and WheatBreadNoWholegrain from children with highest intake of foods such as FruitNutSnack, RyeBread, BreakfastCerealsNoAddSugar and Fish.

**Table 2:** Description of food groups and the percentages of children with intake > 0 g/day

Food group	Description	9 mo, % (n = 307^a^)	18 mo, % (n = 267^a^)	36 mo, % (n = 240^a^)
Porridge	Cereal gruel, porridge; homemade or ready-prepared	98	79	59
BreakfastCereals AddSugar	Sugar puffs and sugary cereals	2	14	23
BreakfastCerealsNoAddSugar	Oatmeal, muesli, cornflakes	14	73	90
WheatBreadWholegrain	Grainy bread, crisp bread	72	93	94
WheatBreadNoWholegrain	White bread, biscuits	76	98	97
RyeBread	Rye bread with and without seeds	75	99	100
PastaRice	Pasta, Rice	62	94	93
Potato	Potatoes boiled, baked, mashed or like potato salad	46	76	64
Fruit	Fresh fruit and berries, fruit porridge/soup/compote; homemade or ready-prepared	100	100	100
Vegetable	All vegetables eaten raw/cooked/mashed alone or in a dish	99	100	100
Fish	All fish and fish products eaten as sandwich spread or in a dish	85	90	92
Meat	All meat and meat products eaten as sandwich spread or in a dish, except poultry and fish	98	100	100
Poultry	All poultry and poultry products eaten as sandwich spread or in a dish	59	74	73
Egg	All egg and egg products eaten as sandwich spread or in a dish	74	98	100
FatsAnimal	Butter, spreadable butter, sauce made from butter	93	98	98
FatsVegetable	Oil, margarine, mayonnaise, remoulade, ketchup, low fat sauce	73	83	90
Cheese	All cheese and cheese products eaten as sandwich spread or in a dish	79	98	96
Milk	All milk and milk products, e.g. skimmed milk, semi-skimmed milk, full fat milk and yogurt, eaten alone or in a dish except human milk or infant formula	98	100	100
Formula	Infant formula	70	3	0
BreastMilk	Human milk from the mother	42	0	0
FruitNutSnack	Cereal bar, nuts, almonds, dried fruit and fruit spread, jam, honey, peanut butter, seeds, peanuts	62	96	98
Chips	Potato chips, popcorn	2	24	37
SweetsCake	Ice cream, chocolate, liquorice, soufflé, croissant, Danish pastry, cookies, cream cake, pancake, cream puff, mix of light/not light versions	27	88	99
SugaryDrink	Soda, juice, lemonade, chocolate milk, milkshake and yogurt drink, mix of light/not light versions	19	76	94
FastFood	Fried potato, French fries, hotdog, pizza, burger, spring rolls	51	88	92

^a^ *BreastMilk*: n_9mo_ = 310, n_18mo_ = 289, n_36mo_ = 270

### 3.3. Categories of development in dietary patterns from 9 to 36 months

The number of children in each category of development in dietary patterns is shown in **Table 3**. The categories have been interpreted based on the naming of each of the three dietary patterns from Figure 3. From this table it is seen that lower adherence to the *Transition Food* pattern than average at all ages (category BBB) was the most common development for this pattern. It was also common to stay either below or above the average of the cohort at all three ages (category BBB or AAA) for the *Healthy Food* and the *Traditional Food* pattern. This indicates tracking of dietary pattern and was further supported by the fact that the observed number of children in category AAA and BBB was higher for all three dietary patterns than the theoretically calculated numbers expected if no tracking was present.

**Table 3:** Categories of individual development in dietary patterns from 9 to 36 months according to the average PCA score at each age in SKOT I (n = 229)

Category	*Transition Food*	n_obs_^a^	n_expt_^b^
**AAA:** Above-Above-Above	More *Transition Food* than average at all ages	24	21
**AAB:** Above-Above-Below	More *Transition Food* than average at 9+18 mo		
	Less *Transition Food* than average at 36 mo	29	-
**ABA:** Above-Below-Above	More *Transition Food* than average at 9+36 mo		
	Less *Transition Food* than average at 18 mo	22	-
**BAA:** Below-Above-Above	More *Transition Food* than average at 18+36 mo		
	Less *Transition Food* than average at 9 mo	26	-
**BBA:** Below-Below-Above	More *Transition Food* than average at 36 mo		
	Less *Transition Food* than average at 9+18 mo	26	-
**ABB:** Above-Below-Below	More *Transition Food* than average at 9 mo		
	Less *Transition Food* than average at 18+ 36 mo	29	-
**BAB:** Below-Above-Below	More *Transition Food* than average at 18 mo		
	Less *Transition Food* than average at 9+36 mo	24	-
**BBB:** Below-Below-Below	Less *Transition Food* than average at all ages	49	39

**Table:** 

Category	*Healthy Food*	n_obs_^a^	n_expt_^b^
**AAA:** Above-Above-Above	More *Healthy Food* than average at all ages	47	29
**AAB:** Above-Above-Below	More *Healthy Food* than average at 9+18mo		
	Less *Healthy Food* than average at 36 mo	23	-
**ABA:** Above-Below-Above	More *Healthy Food* than average at 9+36 mo		
	Less *Healthy Food* than average at 18 mo	22	-
**BAA:** Below-Above-Above	More *Healthy Food* than average at 18+36 mo		
	Less *Healthy Food* than average at 9 mo	33	-
**BBA:** Below-Below-Above	More *Healthy Food* than average at 36 mo		
	Less *Healthy Food* than average at 9+18 mo	14	-
**ABB:** Above-Below-Below	More *Healthy Food* than average at 9 mo		
	Less *Healthy Food* than average at 18+36 mo	17	-
**BAB:** Below-Above-Below	More *Healthy Food* than average at 18 mo		
	Less *Healthy Food* than average at 9+36 mo	17	-
**BBB:** Below-Below-Below	Less *Healthy Food* than average at all ages	56	28

**Table:** 

Category	*Traditional Food*	n_obs_^a^	n_expt_^b^
**AAA:** Above-Above-Above	More *Traditional Food* than average at all ages	32	26
**AAB:** Above-Above-Below	More *Traditional Fod* than average at 9+18 mo		
	Less *Traditional Food* than average at 36 mo	28	-
**ABA:** Above-Below-Above	More *Traditional Food* than average at 9+36 mo		
	Less *Traditional Food* than average at 18 mo	24	-
**BAA:** Below-Above-Above	More *Traditional Food* than average at 18+36 mo		
	Less *Traditional Food* than average at 9 mo	32	-
**BBA:** Below-Below-Above	More *Traditional Food* than average at 36 mo		
	Less *Traditional Food* than average at 9+18 mo	25	-
**ABB:** Above-Below-Below	More *Traditional Food* than average at 9 mo		
	Less *Traditional Food* than average at 18+36 mo	26	-
**BAB:** Below-Above-Below	More *Traditional Food* than average at 18 mo		
	Less *Traditional Food* than average at 9+18 mo	20	-
**BBB:** Below-Below-Below	Less *Traditional Food* than average at all ages	42	31

^a^n: observed number of children in each category. The darkness of grey colour increase with n (Colour cut-off: n < 22, 22–31, 32–41, > 41). The categorisation was based on individual PCA scores of each child as either above or below mean score of the whole SKOT I cohort at the same age. ^b^Theoretically calculated n expected if no tracking was present

### 3.4. Development in the Transition Food pattern related to 36 months outcomes

**Figure 4:**
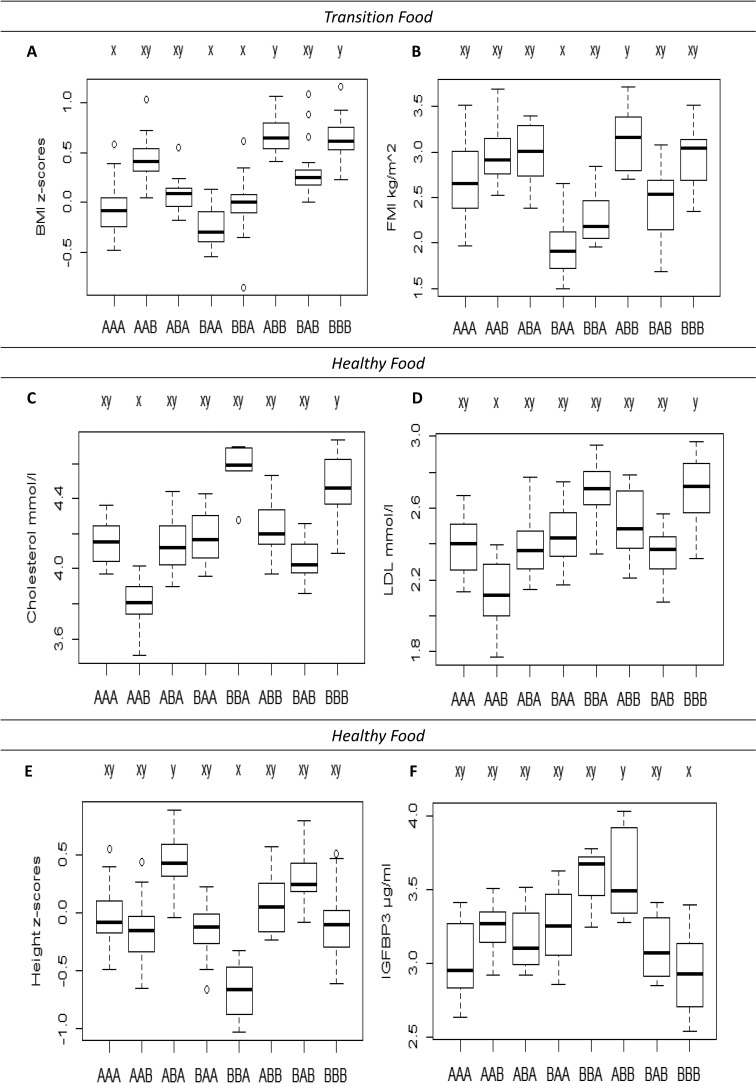
Differences in body size, body composition and metabolic risk markers at 36 months between the categories of development in the dietary patterns *Transition Food* and *Healthy Food*. A, B: BMI z-scores and FMI respectively in the eight categories of development in the *Transition Food* pattern. C, D, E, F: Total blood cholesterol, LDL, height z-scores and IGFBP3 respectively in the eight categories of development in the *Healthy Food* pattern. Pairwise comparisons are based on ANCOVA. Letters below the boxplot (A, B) refer to the different categories of development in dietary patterns (Table 3). Significant different levels in the dietary categories are indicated with different letters (x or y) above the boxplot. Boxes indicate the interquartile range around the median and are extended by lines of +/-1.5 * interquartile range (or maximum/minimum, if these are within 1.5* interquartile range) and single more extreme values are indicated with dots

Mean BMI z-score (p < 0.001) and FMI (p = 0.006) at 36 mo were significantly different between the eight categories of development in the *Transition Food* pattern. The BMI z-scores at 36 mo were higher for children with lower adherence to the *Transition Food* pattern than average at 18 and 36 mo irrespectively of intake at 9 mo (categories ABB, BBB) compared to children with higher adherence to the *Transition Food* pattern than average at 36 mo (categories AAA, BAA, BBA) (**Figure 4**A). The same trend was seen for FMI at 36 mo (Figure 4B), however not significantly for category BBA and BBB. The *Transition Food* pattern was not associated with height z-score, FFMI, glucose, insulin, total cholesterol, HDL, LDL, triacylglycerol, IGF-I, IGFBP3 and IGF-I/IGFBP3 at 36 mo of age. The result in Figure 4 can also be viewed in the supplementary **Table S3**.

### 3.5. Development in the Healthy Food pattern related to 36 months outcomes

The mean height z-score (p = 0.04), IGFBP3 (p = 0.04), total cholesterol (p = 0.02) and LDL (p = 0.03) at 36 mo were significantly different between the eight categories of development in the *Healthy Food* pattern. Significant differences in cholesterol or LDL were seen when the categories of development in the *Healthy Food* pattern varied for 9 and 18 mo irrespectively of the intake at 36 mo. This trend was also observed at 9 mo for height and IGFBP3 irrespectively of intake at 18 and 36 mo. This is based on the following findings. Total cholesterol and LDL were higher for children eating less healthy than average at all three ages (category BBB) compared to children eating more healthy than average at the first two registrations, 9 and 18 mo and less healthy than average at the last registration, 36 mo (category AAB) (Figure 4C, D). The height z-score was lower for children eating less healthy than average at both 9 and 18 mo and more healthy than average at 36 mo (category BBA) compared to children eating more healthy than average at 9 mo (category ABA) (Figure 4E). IGFBP3 at 36 mo was higher for children eating more healthy than average at 9 mo (category ABB) compared to children eating less healthy than average at 9 mo (category BBB) in groups who ate less healthy than average at both 18 and 36 mo (Figure 4F). The *Healthy Food* pattern was not associated with BMI z-score, FMI, FFMI, glucose, insulin, HDL, triacylglycerol, IGF-I and IGF-I/IGFBP3 at 36 mo of age.

### 3.6. Development in the Traditional Food pattern related to 36 months outcomes

None of the tested outcomes (height z-score, BMI z-score, FMI, FFMI, glucose, insulin, total cholesterol, HDL, LDL, triacylglycerol, IGF-I, IGFBP3, and IGF-I/IGFBP3 at 36 mo of age) were associated with the *Traditional Food* pattern.

## 4. Discussion

Three dietary patterns were identified: *Transition Food*, *Healthy Food* and *Traditional Food*. When categorising children according to the course of development in each of these three dietary patterns over ages, the most common course among the children was to stay either above or below the average of the cohort at all three ages. BMI z-score and FMI at 36 mo were significantly different between categories of development in the *Transition Food* pattern, while total blood cholesterol, LDL, height z-score and IGFBP3, at 36 mo differed significantly between the categories of development in the *Healthy Food* pattern. Especially, groups of children with lower adherence to the *Transition Food* pattern or lower adherence to the *Healthy Food* pattern than average at two or all three ages had higher BMI, FMI and metabolic risk markers and thereby seem to represent undesirable development in dietary patterns for toddlers.

### 4.1. Similarities of dietary patterns across populations and studies

As far as we know, only the ALSPAC publication by Brazionis and colleges [12] includes dietary records from different ages in the same PCA to look for development in dietary patterns and is therefore comparable to our study. However, they do not evaluate tracking at the individual level as we do. The main food groups contributing to high scores at our *Healthy Food* pattern (Figure 3A) seem to be comparable to the healthy pattern in ALSPAC [12], and low scores at our *Healthy Food* pattern resemble the less healthy pattern in ALSPAC apart from a distinct contribution of either breast milk or formula in ALSPAC. This distinct contribution of breast milk and formula is probably caused by the timing of the first registration at 6 mo in ALSPAC compared to 9 mo in SKOT I.

Low scores in our *Transition Food* pattern might be related with the infant guideline pattern identified in the SWS cohort for children at 6 and 12 mo, while high scores at our *Transition Food* pattern might be related with their adult food pattern [4]. The SKOT I cohort showed both baby- and family/solid-foods more roughly in one dietary pattern instead of two because our PCA also included 18 and 36 mo diet records.

The naming of the *Traditional Food* pattern is based on a traditional Danish hot dinner with potato, sauce and meat which is represented in high scores for this pattern. Rye bread is also a traditional food for lunch in Denmark but might now be linked with modern life and progress as part of the New Nordic Diet concept [32]. A traditional food pattern has also been reported in other studies, e.g. in a Norwegian study of two year old children. The food cultures across the Scandinavian countries have a lot of similarities and the identified Norwegian traditional food pattern also resembles our pattern of a hot meal with potato, sauce and meat [14]. The *Traditional Food* pattern in SKOT I was not associated with any of the health outcomes even though low loadings at this pattern show similarities with high loadings in the *Healthy Food* pattern like Fish and RyeBread.

### 4.2. Tracking of dietary patterns

A higher proportion of participants in SKOT I kept the same position in PCA scores relative to the cohort mean from 9 mo until our last recording at 36 mo (categories AAA, BBB) than expected if the individual score values were independent over time (Table 3). This was true for all three dietary patterns. Maintaining the same course in dietary patterns at the individual level over time indicates tracking and was also observed in SWS from 6 mo to 12 mo [4]. Tracking of dietary patterns is presumably related to tracking of parental choices and routines at this early age. Tracking beyond 36 mo was seen in a Finnish study of dietary patterns in children 3–18 years of age at baseline with follow-up after 6 and 21 years [3]. This indicates that children already at the age of complementary feeding can be categorised according to a more or less healthy dietary profile which might track for several years. But equally important to note; another group of the participants changed their adherence to the dietary patterns over time (categories AAB, BAA, BBA, ABB and in particular ABA and BAB).

### 4.3. Association between development in dietary patterns and body composition

The *Transition Food* pattern primarily showed an age gradient in the development of diet from baby to family food. However, this pattern might be more than an age gradient because it was also associated with risk markers for later obesity. The unfavourable findings of higher BMI z-scores and FMI for children with lower adherence to the *Transition Food* pattern than average at both 18 and 36 mo (categories ABB, BBB) are difficult to interpret but might be related to a less diversified dietary intake for these children. This is supported by a higher mean intake of Porridge in category ABB and BBB for the *Transition Food* pattern. Porridge is the only food group with low loadings at the *Transition Food* pattern which is eaten by most of the children at 18 and 36 months and therefor relevant here. Moreover, when looking at the cohort in general, children with a higher intake of Porridge at 18 and 36 months, compared to the cohort median, had higher mean BMI z-score at 36 months than children both with a lower intake of Porridge and the whole cohort. Another possibility is a higher degree of under-reporting for children with highest BMI and FMI. However, the estimated proportion of under-reporters is low compared to other studies [33]. We do not know of other studies investigating the association between longitudinal development of dietary patterns and body composition in toddlers. Nevertheless, a few studies investigated the association of dietary patterns at one age during toddlerhood or childhood and current or later body composition. The British SWS [17] and the Australian study [11] with toddlers 12 to 24 mo did not find any association between dietary patterns and current skinfold thickness or BMI z-score, respectively. In a follow-up at 4 years in SWS they investigated whether an association appears later in childhood [34] and found that children eating most similar to infant guidelines at 12 mo had a higher lean mass index compared to children not eating according to infant guidelines, but no difference in FMI or BMI was found. Hereby, time for accumulation of dietary effects might be needed before an effect occurs, which might be a possible explanation for our finding at 36 mo in contrast to no association found in the British and Australian studies earlier in life. A healthy food pattern has been associated with lower BMI or fat mass in older children, 3–18 years of age [35]–[37]. In addition, including the whole profile of development in dietary patterns at 9, 18 and 36 mo in our analysis might contribute to making this early accumulating effect more clear.

### 4.4. Association between development in dietary patterns and metabolic risk markers

To our knowledge, no studies compare dietary patterns with biomarkers in children below 5 years, as was also pointed out in a recent review [9], and few such studies have been carried out with children above 5 years and adolescents. We found an association between the *Healthy Food* pattern and the potential metabolic risk markers of later cardiovascular diseases, LDL and total cholesterol. Intake during the early period (9, 18 mo) tended to be most important for these associations because categories AAB and BBB differed at 9 and 18 mo, but not at 36 mo of age. The association between higher total cholesterol and LDL, and potentially unfavourable dietary patterns, found in SKOT I, was also seen in a Finnish study of children 3–18 years old [35] and among Chinese children aged 6–13 years [36]. These associations are biological plausible based on nutrients involved in the mechanism regulating the blood LDL level in toddlerhood [38]. Considerable amounts of fibre, monounsaturated, and polyunsaturated fat are found in the foods with highest loadings (Vegetable, Fruits, Fish, and FatsVegetable) in the *Healthy Food* pattern and might contribute to lower LDL levels, while saturated fat and sugar are highly present in foods with lowest loadings (SugaryDrink and SweetsCake) and might contribute to higher LDL levels. In addition, the Finish and Chinese studies also found associations between dietary patterns and triacylglycerol, insulin, glucose and HDL, while we did not find any such associations.

### 4.5. Association between development in dietary patterns, height and IGFBP3

The association between the *Healthy Food* pattern and height in SKOT I cannot directly be confirmed by other studies examining dietary patterns, as only few studies have looked at this. SWS did not find any association between dietary patterns and height at 12 mo [17]. However, the relation between dietary patterns and height might be more pronounced in food insecure settings compared to affluent cohorts like SWS or SKOT I. A case-control study comparing 7-year-old stunted and non-stunted children in Iran found that children with the highest intake of a carbohydrate-protein pattern, including highest intake of sweets, meat and dairy products, tended to be less likely to be stunted compared to children with the lowest intake [39]. We found in a previous cross sectional study of 2.5-year-old Danish children, where we did not examine dietary patterns, that height was positively associated with milk, but not with meat intake [40]. In SKOT I the milk intake is high compared to other food groups (Figure 2) and in the *Healthy Food* pattern milk has a positive although small loading (Figure 3A), which might be one of the reasons for the positive association with height. Especially, the diet at 9 mo seems important for the association with height when compared with 18 and 36 mo (category ABA versus BBA). The difference between the category with the lowest IGFBP3 (category BBB) and highest IGFBP3 (category ABB) for the *Healthy Food* pattern also indicated importance of 9 mo patterns. IGF-I is important for linear growth during childhood [41] and the binding protein IGFBP3 regulates the level of active IGF-I and is therefore assumed to be negatively associated with growth, opposite to IGF-I [41]. However, we have in previous studies found that both IGF-I and IGFBP3 and the ratio IGF-I/IGFBP3 are stimulated by certain foods and diets (e.g. milk intake) in children [42]. Thus, milk intake might also contribute to the association between the *Healthy Food* pattern and IGFBP3, which may reflect a more general effect on the IGF system. To our knowledge, we are the first to investigate associations between IGF-1, the binding protein, and dietary patterns in childhood.

### 4.6. Strengths and limitations

The main strengths in our study are the longitudinal approach, not only looking at cross sectional dietary patterns, and the use of seven days' food diaries with thorough portion size estimations. Including longitudinal data in one PCA ensures that score values from different time points are compared precisely on the same dietary pattern; this approach is widely accepted in other fields using PCA [43];[44] but has only been validated sparingly within child nutrition [12]. Moreover, investigating the link to body size, body composition and metabolic risk markers strengthens the study.

It is a general limitation that PCA is not independent of subjective decisions when selecting content and number of food groups even though it is a data-driven method. This is especially worth noticing when condensing the dietary complexity extensively as we do in this study both across the whole diet and across different ages. We ended up with relatively few and broad dietary variables in the PCA to ease the visualisation and interpretation of data. Our relatively precise portion size estimation of different food groups is probably as important as differentiating between very specific food groups when looking at associations with body composition and risk markers of later diseases. Moreover, the categorisation method for development in dietary patterns used in this paper is not a validated method, which might be considered as another limitation and should be further investigated. The categorisation suggested here is one way of simplifying types of developments. Other more model based approaches including linear mixed models can be applied to address tracking [35];[45]. However, this type of modelling induces a very particular type of tracking the validity of which needs close scrutiny. As our aim has been a mere description of potential tracking without imposing an array of potentially unrealistic assumptions we have deferred from using these modelling approaches. Our method has the advantage that it enables the inclusion of three time points in one simple variable describing the course of development in dietary patterns. In addition, our method focuses on archetypes rather than potentially less important minor individual differences. Lastly, these findings might not be representative for Danish children because the SKOT I cohort is a relatively small, relatively homogeneous group with parents having a high socioeconomic status and all living in the capital area. The socioeconomic status in SKOT I has previously been compared with another Danish cohort [46].

## 5. Conclusion

Development in dietary patterns can, in an informative manner, be characterised by PCA and related to toddlerhood body size, body composition and metabolic risk markers. The explorative method applied in this analysis seems to be able to handle some of the complexity in the understanding of child nutrition. The tracking of dietary patterns already from 9 mo for some children, together with changes in the course of development in dietary patterns for other children and the association with risk markers, even within a high socioeconomic and relatively homogeneous population, emphasize the need for early and sustained promotion of healthy diets in the first years of life.

## Key Messages

Tracking of dietary patterns was observed already from 9 mo for a relative large group of children, together with changes in the course of development in dietary patterns for other children.

Development of certain dietary patterns from 9 to 36 mo was associated with potential risk markers of later obesity and cardiovascular diseases.

These findings, even within a high socioeconomic and relatively homogeneous population, emphasise the need for early and sustained promotion of healthy diets in the first years of life.

## Figures and Tables

**Table S1:** Loadings for input variables in the PCA based on dietary intake at 9, 18 and 36 months of age for children from the SKOT I cohort

	Dietary patterns (% variance explained)		
Foods (input variables for PCA)^a^	*Transition Food* (18%)	*Healthy Food* (8%)	*Traditional Food* (5%)
Porridge (g/kg BW/d)	-0.276	0.249	0.140
BreakfastCerealsAddSugar (g/kg BW/d)	0.100	-0.045	0.072
BreakfastCerealsNoAddSugar (g/kg BW/d)	0.208	-0.081	-0.276
WheatBreadWholegrain (g/kg BW/d)	0.122	0.151	-0.162
WheatBreadNoWholegrain (g/kg BW/d)	0.250	-0.021	0.271
RyeBread (g/kg BW/d)	0.234	0.172	-0.268
PastaRice (g/kg BW/d)	0.202	0.168	0.027
Potato (g/kg BW/d)	0.107	0.212	0.399
Fruit (g/kg BW/d)	-0.039	0.426	-0.014
Vegetable (g/kg BW/d)	0.068	0.326	-0.166
Fish (g/kg BW/d)	0.074	0.334	-0.263
Meat (g/kg BW/d)	0.284	0.123	0.182
Poultry (g/kg BW/d)	0.070	0.174	-0.122
Egg (g/kg BW/d)	0.257	-0.093	0.046
FatsAnimal (g/kg BW/d)	0.132	0.254	0.494
FatsVegetable (g/kg BW/d)	0.071	0.254	-0.052
Cheese (g/kg BW/d)	0.166	0.197	-0.105
Milk (g/kg BW/d)	0.282	0.068	0.116
Formula (g/kg BW/d)	-0.281	0.085	-0.004
BreastMilk (feedings/d)	-0.222	-0.044	0.204
FruitNutSnack (g/kg BW/d)	0.221	-0.049	-0.279
Chips (g/kg BW/d)	0.152	-0.181	0.075
SweetsCake (g/kg BW/d)	0.280	-0.289	0.039
SugaryDrink (g/kg BW/d)	0.231	-0.221	-0.001
FastFood (g/kg BW/d)	0.245	0.030	0.152

BW: body weight, ^a^n_9mo_=307, n_18mo_=267, n_36mo_=240, except for *BreastMilk*: n_9mo_=310, n_18mo_=289, n_36mo_=270

**Table S2:** Mean scores according to age for each dietary pattern identified in a PCA based on dietary intake at 9, 18 and 36 months of age for children from the SKOT I cohort

Age	*Transition Food* mean ±SD (n)	*Healthy Food* mean ±SD (n)	*Traditional Food* mean ±SD (n)
9 mo	-2.2±1.3(307)	0.30±1.42(307)	0.25±0.95(307)
18 mo	1.4±1.3(267)	0.38±1.41(267)	0.15±1.36(267)
36 mo	1.3±1.0(240)	-0.81±1.17(240)	-0.49±0.98(240)

**Table S3:** Differences in body size, body composition and metabolic risk markers at 36 months between categories of development in the dietary patterns *Transition Food* and *Healthy Food*

Compared	*Transition Food*						*Healthy Food*											
	BMI z-score			FMI, kg/m^2^			Total Cholesterol, mmol/l			LDL, mmol/l			Height z-score			IGFBP3, µg/ml		
categories^a^	Est. diff.^b^	95% CI^c^	p^d^	Est. diff.	95% CI	p	Est. diff.	95% CI	p	Est. diff.	95% CI	p	Est. diff.	95% CI	p	Est. diff.	95% CI	p
AAB-AAA	0.55	-0.17, 1.27	0.28	0.30	-0.61, 1.21	0.97	-0.41	-1.02, 0.20	0.43	-0.33	-0.85, 0.19	0.52	-0.11	-0.80, 0.57	1.0	0.076	-0.53 0.68	1.0
ABA-AAA	0.12	-0.64, 0.88	1.0	0.13	-0.96, 1.22	1.0	0.0086	-0.60, 0.61	1.0	-0.00025	-0.52, 0.52	1.0	0.41	-0.26, 1.09	0.56	0.17	-0.43 0.76	1.0
BAA-AAA	-0.13	-0.85, 0.58	1.0	-0.63	-1.69, 0.43	0.58	0.039	-0.50, 0.58	1.0	0.076	-0.39, 0.542	1.0	-0.11	-0.71, 0.48	1.0	0.19	-0.34 0.71	0.96
BBA-AAA	0.070	-0.682, 0.823	1.0	-0.30	-1.35, 0.74	0.98	0.42	-0.36, 1.21	0.72	0.28	-0.39, 0.96	1.0	-0.65	-1.50, 0.21	0.28	0.42	-0.35 1.19	0.69
ABB-AAA	0.78	0.07, 1.50	***0.021***	0.49	-0.52, 1.49	0.80	0.10	-0.54, 0.74	1.0	0.15	-0.40, 0.70	0.99	0.13	-0.61, 0.86	1.0	0.57	-0.05 1.20	0.097
BAB-AAA	0.40	-0.33, 1.14	0.70	-0.49	-1.54, 0.56	0.82	-0.094	-0.82, 0.63	1.0	0.00094	-0.62, 0.62	1.0	0.25	-0.51, 1.01	0.97	0.050	-0.64 0.74	1.0
BBB-AAA	0.72	0.07, 1.36	***0.017***	0.24	-0.74, 1.21	0.99	0.30	-0.18, 0.78	0.55	0.27	-0.14, 0.69	0.46	0.0025	-0.52, 0.53	1.0	-0.11	-0.58 0.36	1.0
ABA-AAB	-0.43	-1.17, 0.31	0.63	-0.17	-1.15, 0.80	1.0	0.42	-0.27, 1.10	0.56	0.33	-0.26, 0.92	0.66	0.53	-0.26, 1.31	0.44	0.093	-0.58 0.77	1.0
BAA-AAB	-0.68	-1.37, 0.01	0.053	-0.93	-1.88, 0.02	0.059	0.45	-0.19, 1.09	0.38	0.41	-0.14, 0.96	0.31	-0.00035	-0.72, 0.72	1.0	0.11	-0.51 0.74	1.0
BBA-AAB	-0.48	-1.20 0.23	0.44	-0.61	-1.51, 0.30	0.42	0.83	-0.01, 1.67	0.056	0.61	-0.11, 1.34	0.16	-0.53	-1.46, 0.39	0.64	0.35	-0.48 1.18	0.90
ABB-AAB	0.23	-0.46, 0.93	0.97	0.18	-0.74, 1.10	1.0	0.51	-0.21, 1.24	0.37	0.48	-0.14, 1.10	0.26	0.24	-0.59, 1.08	0.99	0.50	-0.22 1.21	0.39
BAB-AAB	-0.15	-0.86, 0.56	1.0	-0.79	-1.74, 0.15	0.17	0.32	-0.49, 1.12	0.93	0.33	-0.36, 1.02	0.82	0.36	-0.50, 1.22	0.90	-0.026	-0.80 0.74	1.0
BBB-AAB	0.17	-0.45, 0.79	0.99	-0.067	-0.90, 0.77	1.0	0.71	0.12, 1.30	***0.0075***	0.60	0.10, 1.11	***<0.01***	0.12	-0.54, 0.78	1.0	-0.19	-0.77 0.39	0.97
BAA-ABA	-0.25	-0.99, 0.48	0.96	-0.76	-1.86, 0.34	0.39	0.030	-0.60, 0.66	1.0	0.076	-0.47, 0.62	1.0	-0.53	-1.25, 0.19	0.33	0.018	-0.60 0.64	1.0
BBA-ABA	-0.051	-0.80, 0.70	1.0	-0.43	-1.49, 0.63	0.90	0.41	-0.42, 1.25	0.79	0.28	-0.43, 1.0	0.93	-1.061	-1.99, -0.13	***0.014***	0.25	-0.56 1.07	0.98
ABB-ABA	0.66	-0.07, 1.40	0.11	0.36	-0.74, 1.46	0.97	0.094	-0.64, 0.83	1.0	0.15	-0.48, 0.78	1.0	-0.28	-1.13, 0.56	0.97	0.41	-0.31 1.13	0.66
BAB-ABA	0.28	-0.48, 1.04	0.95	0.62	-1.69, 0.45	0.61	-0.10	-0.90, 0.69	1.0	0.0012	-0.68, 0.68	1.0	-0.16	-1.03, 0.70	1.0	-0.12	-0.89 0.65	1.0
BBB-ABA	0.60	-0.07, 1.26	0.11	0.11	-0.90, 1.11	1.0	0.29	-0.29, 0.87	0.78	0.27	-0.22, 0.77	0.69	-0.41	-1.07, 0.25	0.54	-0.28	-0.85 0.29	0.79
BBA-BAA	0.20	-0.50, 0.90	0.99	0.32	-0.70, 1.35	0.97	0.38	-0.41, 1.18	0.82	0.21	-0.47, 0.89	0.98	-0.53	-1.41, 0.34	0.57	0.24	-0.54 1.02	0.98
ABB-BAA	0.92	0.23, 1.60	***<0.01***	1.1	0.07, 2.16	***0.029***	0.064	-0.61, 0.73	1.0	0.072	-0.50, 0.65	1.0	0.24	-0.54, 1.02	0.98	0.39	-0.27 1.04	0.60
BAB-BAA	0.53	-0.18, 1.25	0.30	0.14	-0.94, 1.21	1.0	-0.13	-0.88, 0.62	1.0	-0.075	-0.72, 0.57	1.0	0.36	-0.44, 1.16	0.86	-0.14	-0.85 0.57	1.0
BBB-BAA	0.85	0.24, 1.46	***<0.01***	0.87	-0.12, 1.85	0.13	0.26	-0.26, 0.78	0.79	0.20	-0.25, 0.65	0.87	0.12	-0.47, 0.70	1.0	-0.30	-0.80 0.21	0.60
ABB-BBA	0.71	0.01, 1.41	***0.042***	0.79	-0.19, 1.78	0.21	-0.32	-1.20, 0.56	0.95	-0.13	-0.89, 0.62	1.0	0.78	-0.21, 1.76	0.24	0.15	-0.71 1.02	1.0
BAB-BBA	0.33	-0.41, 1.07	0.86	-0.19	-1.20, 0.83	1.0	-0.52	-1.45, 0.41	0.68	-0.288	-1.08, 0.52	0.96	0.90	-0.091, 1.89	0.11	-0.37	-1.27 0.52	0.90
BBB-BBA	0.65	0.02, 1.28	***0.039***	0.54	-0.33, 1.42	0.54	-0.12	-0.87, 0.63	1.0	-0.0096	-0.65, 0.63	1.0	0.65	-0.17, 1.48	0.24	-0.53	-1.27 0.20	0.33
BAB-ABB	-0.38	-1.10, 0.33	0.72	-0.98	-2.02, 0.07	0.082	-0.20	-1.03, 0.63	1.0	-0.15	-0.86, 0.57	1.0	0.12	-0.79, 1.03	1.0	-0.53	-1.32 0.27	0.46
BBB-ABB	-0.066	-0.67, 0.54	1.0	-0.25	-1.17, 0.67	0.99	0.20	-0.43, 0.82	0.98	0.13	-0.41, 0.66	1.0	-0.13	-0.85, 0.59	1.0	-0.69	-1.30 -0.070	***0.018***
BBB-BAB	0.32	-0.33, 0.96	0.80	0.73	-0.23, 1.68	0.27	0.39	-0.32, 1.11	0.69	0.27	-0.34, 0.88	0.87	-0.25	-1.00, 0.51	0.97	-0.16	-0.84 0.51	1.0

^a^Representing pairwise comparison of the eight categories of development in dietary patterns across 9, 18 and 36 mo of life, A=PCA score Above the mean score for each age, B= PCA score Below the mean score for each age. ^b^Estimated difference in the outcome between the two categories compared. ^c^Familywise 95% confidence interval adjusted for multiple testing. ^d^Post hoc pairwise comparisons of the eight categories adjusted for multiple testing using the single step method. P-values below 0.05 are indicated with bold numbers.
